# Chitosan-Based Membranes as Gentamicin Carriers for Biomedical Applications—Influence of Chitosan Molecular Weight

**DOI:** 10.3390/membranes13060542

**Published:** 2023-05-23

**Authors:** Milena Supernak, Balbina Makurat-Kasprolewicz, Beata Kaczmarek-Szczepańska, Anna Pałubicka, Monika Sakowicz-Burkiewicz, Anna Ronowska, Marcin Wekwejt

**Affiliations:** 1 Institute of Naval Architecture and Ocean Engineering, Gdańsk University of Technology, 80-233 Gdańsk, Poland; 2 Department of Materials Science and Technology, Faculty of Mechanical Engineering and Ship Technology, Gdańsk University of Technology, 80-233 Gdańsk, Poland; 3 Department of Biomaterials and Cosmetics Chemistry, Faculty of Chemistry, Nicolaus Copernicus University in Torun, 87-100 Toruń, Poland; 4 Department of Laboratory Diagnostics and Microbiology with Blood Bank, Specialist Hospital in Kościerzyna, 83-400 Kościerzyna, Poland; 5 Departament of Molecular Medicine, Medical University of Gdańsk, 80-210 Gdańsk, Poland; 6 Department of Laboratory Medicine, Medical University of Gdańsk, 80-210 Gdańsk, Poland; 7 Department of Biomaterials Technology, Faculty of Mechanical Engineering and Ship Technology, Gdańsk University of Technology, 80-233 Gdańsk, Poland

**Keywords:** chitosan, membrane, gentamicin sulfate, infection treatment, antibacterial activity, cytocompatibility, drug carrier

## Abstract

Over the past decade, much attention has been paid to chitosan as a potential drug carrier because of its non-toxicity, biocompatibility, biodegradability and antibacterial properties. The effect of various chitosan characteristics on its ability to carry different antibiotics is discussed in the literature. In this work, we evaluated the influence of the different molecular weights of this polymer on its potential as an antibacterial membrane after adding gentamicin (1% *w*/*w*). Three types of chitosan membranes without and with antibiotic were prepared using a solvent casting process. Their microstructures were analyzed with a 4K digital microscope, and their chemical bonds were studied using FTIR spectroscopy. Furthermore, cytocompatibility on human osteoblasts and fibroblasts as well as antibacterial activity against *Staphylococcus aureus* (*S. aureus.*) and *Escherichia coli* (*E. coli*) were assessed. We observed that the membrane prepared from medium-molecular-weight chitosan exhibited the highest contact angle (≈85°) and roughness (10.96 ± 0.21 µm) values, and its antibacterial activity was unfavorable. The maximum tensile strength and Young’s modulus of membranes improved and elongation decreased with an increase in the molecular weight of chitosan. Membranes prepared with high-molecular-weight chitosan possessed the best antibacterial activity, but mainly against *S. aureus*. For *E. coli*, is not advisable to add gentamicin to the chitosan membrane, or it is suggested to deplete its content. None of the fabricated membranes exhibited a full cytotoxic effect on osteoblastic and fibroblast cells. Based on our results, the most favorable membrane as a gentamicin carrier was obtained from high-molecular-weight chitosan.

## 1. Introduction

Chitosan is a semi-crystalline polysaccharide derived from chitin, and is known as a broad-spectrum biomaterial [1,2]. In its chemical structure, residues of N-acetyl-d-glucosamine and d-glucosamine can be distinguished [2,3]. Moreover, two kinds of functional groups in chitosan can be identified: an amino group which causes the cationic nature of the compound, and a hydroxyl group which enhances the bond. The presence of the abovementioned functional groups facilitates the uncomplicated modification of chitosan [2].

Chitosan has a distinct origin. It can be manufactured from shrimps, crabs and some crawfish, but it is also present in some species of insects, fungi and yeast [1,3]. Chitosan produced from chitin of different sources may have various physical properties, e.g., a polymer obtained from snail and crab shells differs in molecular weight, among other properties [4,5,6]. The viscosity, crystallinity and solubility of the polymer depend inversely on the degree of deacetylation and the molecular mass of polysaccharide achieved during the process [7]. However, each type of chitosan exhibits biodegradability, biocompatibility, non-toxicity and hydrophilicity [8]. Furthermore, chitosan is a chelating agent and possesses antibacterial properties [1] because, as a cationic compound, it has an electrostatic effect on the bacterial cell envelope and affects its activity [7]. 

Chitosan is a particularly distinguished biopolymer due to its ability to create various morphological structures, such as films, fibers, hydrogels, membranes, nanoparticles and microgranules [4,9]. The sensitivity of chitosan to pH, non-toxicity, high biocompatibility, biodegradability and antimicrobial properties makes chitosan an essential candidate for many medical applications, including the designing of scaffolds for cartilage tissue, membranes for bone regeneration, artificial skin, wound dressings, drugs and others [4,10,11,12]. Additionally, chitosan membranes are applied for guided bone regeneration (GBR) in dental implantology and scaffolds in guided tissue regeneration (GTR) for producing artificial skin and cartilage [11,13]. Moreover, it is an important antimicrobial agent in wound dressing in hydrogels, xerogels, powders, composites, films and scaffolds [14], as well as antimicrobial leather materials with antimicrobial activity and film-forming capacity [11,15,16]. Chitosan has been proposed as a support material for controlled drug and non-viral gene delivery, cell culture, cancer treatment and tissue engineering [1,16].

The molecular weight of chitosan is its characteristic parameter; it defines its proper-ties. An important role is played by the chitosan production process, which includes an acid treatment (decalcification), an alkaline treatment (deproteinization) and a deinking step. A new alkaline treatment is then applied to produce chitin deacetylation [17]. In this process, there are many factors that can change the properties of the resulting chitosan, such as alkali concentration, incubation time, chitin to alkali ratio, temperature, atmosphere, chitin source [18]. Commercially available chitosan typically has a degree of deacetylation (DD) in the range of 70–95% and a molecular weight in the range of 104–106 gmol^−1^ [19]. This makes the available products characterized by a wide range of properties that define its applications and affect the final utility of the biopolymer.

In recent years, many researchers have addressed the topic of drug release from chitosan-containing systems. pH-neutral chitosan membranes were used to release several non-steroidal therapeutic agents: diclofenac, ibuprofen and ketoprofen. As a result, it was found that these forms of chitosan are promising carriers for both water-soluble and water-insoluble drugs [20]. In another work, Thacharodi et al. [21] proposed a drug delivery system based on cross-linking chitosan for transdermal application, and their studies showed that controlled release of a model drug was possible. Currently, new inorganic–organic drug release membranes are based on the formation of a network of interpenetrating silicates and organic chitosan [22]. In addition, the concept of a functionally graded membrane (FGM) has emerged, in which the drug was successfully released from frozen gelled chitosan matrices at different percentages of glutaraldehyde [23]. The origin and method of production of chitosan affect its properties; hence, İlk et al. [24] studied quercetin release from an original intact material. It was found that three-dimensional chitosan lenses obtained from *Tabanus bovinus* compound eyes could be effectively used as a drug carrier. In another paper, ionically crosslinked chitosan membranes were prepared and the release of 1,4-naphthoquinone was investigated. It was emphasized that some properties of the chitosan membranes (such as molecular structure, porosity and hydrophilicity) influence drug release behavior [25]. Accordingly, it is the subject of ongoing work to determine the effect of the chitosan molecular weight on the properties of membranes and thus its ability to carry antibiotics, such as gentamicin.

The authors of this article focused mainly on surface, mechanical and biological properties of membranes prepared from chitosan of different molecular weights. Cytocompatibility and antibacterial properties are discussed in detail, providing a rich set of information, which are required for membranes with applications in the biological field. Furthermore, basic investigations were carried out to evaluate membrane amendments depending on the selected chitosan. For this purpose, the microstructure, wettability, chemical structure and mechanical properties were investigated. These are significant properties when choosing the distinctive application of the membrane (e.g., as a drug carrier, implant coating or wound dressing), for which the requirements differ.

## 2. Materials and Methods

### 2.1. Membrane Preparation

The chitosan membranes were prepared for each test based on a mixture of chitosan (0.1 g), acetic acid of analytical purity (50 µL) and distilled water (9.85 mL). Three types of chitosan were evaluated: low molecular weight (ChitL; 2–300 cps; 50–190 kDa; 75–85% deacetylated), medium molecular weight (ChitM; 200–800 cps; 190–310 kDa; 75–85% deacetylated) and high molecular weight (ChitH; 800–2000 cps; 310–375 kDa; 75–85% deacetylated). Reagents, unless otherwise noted, were purchased from Merck KGaA (Darmstadt, Germany). The mixture was stirred using a magnetic device to dissolve the chitosan powder completely. After this step, the antibiotic—gentamicin sulfate (G; Sigma Aldrich, Steinheim, Germany)—was added in a proportion of 1% *w*/*w* to the mixed chitosan solution. Next, the homogenization of the solution was made in a laboratory centrifuge at 5000 rev/min for one h. Constant portions of homogenous solution were poured into 6- and 24-well plates and then dried in an incubator at 37 °C for 24 h. In the next stage, the neutralization of membranes was performed using 0.5 M NaOH solution of analytical purity. The hydroxide solution was poured onto membrane surfaces and specimens were placed in the shaker for 30 min. After removal of the excess of neutralizing solution, the membranes were again put into the incubator for 12 h at 37 °C to dry the specimens. Then, sterilization was carried out for 30 min using 70% ethanol of analytical purity poured on membrane surfaces and UV-light. The preparation of membranes was carried out separately for each form of chitosan. The following types of membranes were made: low-molecular-weight chitosan (ChitL), medium-molecular-weight chitosan (ChitM), high-molecular-weight chitosan (ChitH), low-molecular-weight chitosan with gentamicin (ChitL+G), medium-molecular-weight chitosan with gentamicin (ChitM+G) and high-molecular-weight chitosan with gentamicin (ChitH+G).

### 2.2. Microstructure Analysis

The chitosan membrane surfaces were examined with the VHX 7000 digital 4K microscope (VHX-7000, Keyence, Osaka, Japan). Three-dimensional images and surface roughness measurements were obtained with a dedicated Keyence application. The roughness of each membrane was measured linearly, both vertically and horizontally, and the measuring length was 50 µm. 

### 2.3. Wettability

Wettability was assessed by measuring the contact angle of distilled water (n = 5) with the falling drop method at room temperature using an optical tensiometer Attention Theta Life (Biolin Scientific, Espoo, Finland).

### 2.4. Mechanical Properties

Static tensile tests were performed on a universal testing machine Z.05 (Zwick/Roell, Ulm, Germany) with an initial force of 0.1 N and a loading rate of 5 mm/min until the specimens (n = 5) were broken. Maximum tensile strength, Young’s modulus and elongation at the break were determined. The measurement was carried out under room conditions. Specimens were paddle-shaped, were 2.5 cm in length and 0.053 ± 0.007 mm thick.

### 2.5. Chemical Structure

Fourier transform infrared spectroscopy—attenuated total reflectance (FTIR–ATR) spectra were made for each scaffold in the range 4000–600 cm^−1^ using a spectrometer (Nicolet iS5 (Thermo Fisher Scientific, Waltham, MA, USA) equipped with a ZnSe crystal. Spectra were recorded via 64 scans with a resolution of 4 cm^−1^ in absorbance mode. The scale of spectra was normalized using OMNIC software.

### 2.6. Cytocompatibility

The experiments of in vitro cytocompatibility of obtained membranes were conducted on human osteoblast cell line (hFOB 1.19, RRID: CVCL3708; ATCC) and primary human dermal fibroblasts (hDF). Reagents, unless otherwise noted, were purchased from Merck KGaA (Darmstadt, Germany). Human FOB cells were cultured in a 1:1 mixture of Ham’s F12 Medium and Dulbecco’s Modified Eagle’s Medium (without phenol red), supplemented with 0.3 mg/mL geneticin (G418) and 10% Fetal Bovine Serum at 34 °C in a humidified atmosphere with 5% CO_2_. hDF cells were isolated from the human skin during routine plastic surgery. The small pieces of skin were acquired by a physician under approved hospital regulatory protocols. The samples were placed in tubes containing 8–10 mL of ice-cold Dulbecco’s Modified Eagle Medium (DMEM) and immediately transported to the laboratory for cell isolation. The skin samples were washed twice in phosphate-buffered saline (PBS) and they were cut into small pieces and digested with 0.25% Trypsin solution in the growth medium without FBS for 3–4 h. Then, the suspension was filtered with a sterile 100 μM filter into a sterile conical tube to remove debris. The cells were centrifuged at 1200 rpm/10 min, suspended in the complete growth medium containing DMEM, 20% FBS, penicillin (100 U/mL) and streptomycin (0.1 mg/mL), and placed on 6 cm plates. The hDF cells were cultured at 37 °C in a humidified atmosphere with 5% CO_2_. The ethical protocol of the research was approved by the Regional Bioethical Commission at the Medical University of Gdańsk (permission NKBBN/715/2020). The hFOB cells were seeded directly on the membranes, while fDF cells were incubated in a culture plate with membranes for 72 h. Then, cell viability was evaluated using an MTT assay (3-(4,5-dimethylthiazol-2-yl)-2,5-diphenyltetrazolium bromide). The culture medium was withdrawn and replaced with a fresh one containing 0.60 mmol/L of MTT. The culture was continued for 4 h. After incubation, the formazan crystals that had formed were dissolved in solubilizing agent containing 10% sodium dodecyl sulfate and 50% dimethylformamide. Reduced chromophore was determined by spectrophotometric measurement at 570 nm. Treated-cell viability was calculated and expressed as a percentage of viability of untreated control cells—TCP (100%) based on mean absorbance values at 570 nm. 

### 2.7. Antibacterial Properties

Two studies of antibacterial properties were carried out: (1) the zone of inhibition test by the Kirby–Bauer method [26] and (2) the inhibition of bacterial growth in broth by the McFarland method [9]. Briefly, in the first test, 100 μL of the bacterial suspension with inoculum 1.5 × 10^8^ CFU/mL was seeded on Mueller–Hinton agar plates and two different bacterial strains were used: *Staphylococcus aureus* (ATCC 25923) and *Escherichia coli* (ATCC 25922). Next, membranes (diameter 17 mm and thickness 0.05 mm; n = 3) were placed on the prepared bacteria plates and incubated at 37 °C for seven days—zone measurements (±0.1 mm) were made after 1, 3 and 7 days. The second test was based on the optical density of bacterial suspensions. *Staphylococcus aureus* bacteria (ATCC 29213) or *Escherichia coli* (ATCC 25922) with an initial concentration of 0.5 iMS were suspended in Trypticase Soy Broth (2.0 mL; Merck KGaA, Darmstadt, Germany). Then, the broth was added to the wells of 24-well plates containing membranes (thickness 0.05 mm; n = 3) and incubated at 36 °C. After each hour, the optical density was measured using the DensiChEK Plus (BioMerieux, Montreal, QC, Canada). Bacteria incubated without specimens were used as a control. The maximum measuring range of this device was 4 iMS.

### 2.8. Statistical Analysis

Statistical analysis of the data was performed using commercial software (SigmaPlot 14.0, Systat Software, San Jose, CA, USA). The Shapiro–Wilk test was used to assess the normal distribution of the data. All of the results were presented as mean ± standard deviation (SD) and were statistically analyzed using one-way analysis of variance (one-way ANOVA). Multiple comparisons versus the control group between means were performed using the Bonferroni *t*-test with statistical significance set at *p* < 0.05.

## 3. Results

### 3.1. Membrane Preparation

With the increase in molecular weight, the viscosity of the chitosan solutions increased, which resulted in a decrease in their fluidity; consequently, pouring the solution into the mold was more difficult. On the other hand, the use of the low-molecular-weight chitosan solutions contributed to the problematic production of a uniform membrane. No qualitative effect of gentamicin on the membrane preparation process was observed. All obtained membranes were transparent and in a circular or square shape depending on the form in which the solution was placed.

### 3.2. Microstructure Analysis

**Figure 1:**
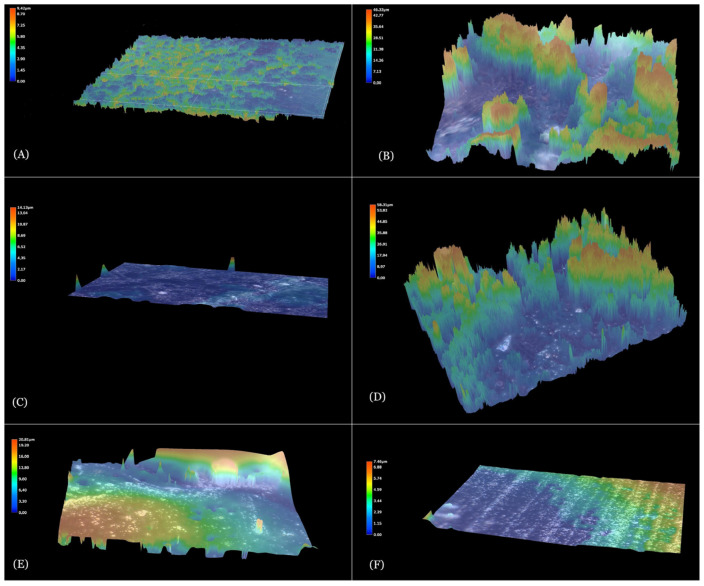
Topography of membranes with: (**A**) ChitL, (**B**) ChitM, (**C**) ChitH, (**D**) ChitL+G, (**E**) ChitM+G, (**F**) ChitH+G. (Magnification 2000×). The membranes exhibit a similar topography; however, for ChitM and ChitM+G surfaces, heterogeneity is observed.

The membranes showed a similar topography. Only in the membrane made of medium-molecular-weight chitosan could greater surface diversity be observed (Figure 1A–C). This may result from the solubility of the powder in acetic acid. The addition of gentamicin subtly affected its diversity, and differences in the films’ roughness were observed (Figure 1D,E).

On the basis of the observations of the topography of membranes, the roughness coefficient was determined for each membrane, as shown in Table 1.

### 3.3. Wettability

**Figure 2:**
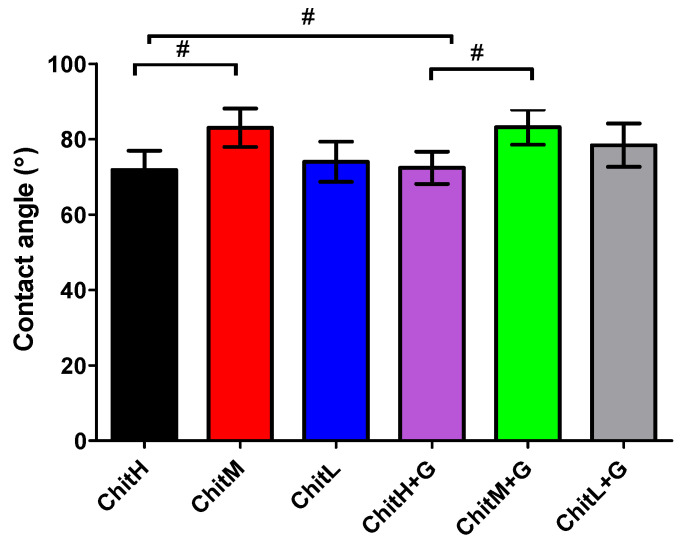
Wettability of membranes determined by water contact angle (n = 5; # statistical significance between groups, *p* < 0.05). All membranes were hydrophilic in nature.

All obtained membranes were hydrophilic and their water contact angle ranged from ≈65 to 85° (Figure 2). It was observed that membranes based on the high-molecular-weight chitosan had lower contact angles than those obtained from medium- and low-molecular weight chitosan. The addition of the antibiotic did not show any significant effect on the wettability of the obtained biomaterials.

### 3.4. Fourier Transform Infrared Spectroscopy—Attenuated Total Reflectance 

**Figure 3:**
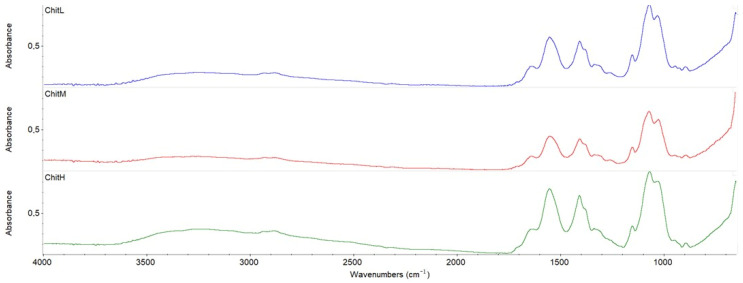
The FTIR spectra of chitosan membranes with different molecular weights. ChitL—membrane with low molecular weight chitosan, ChitM—membrane with medium molecular weight chitosan, ChitH—membrane with high-molecular-weight chitosan.

**Figure 4:**
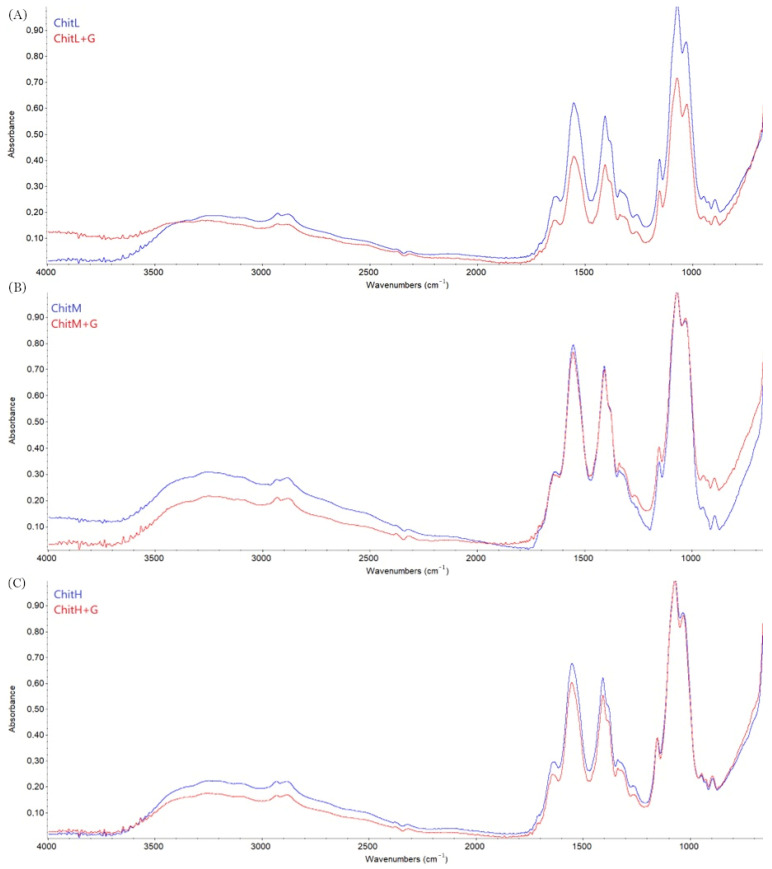
The FTIR spectra of (**A**) ChitL and ChitL+G, (**B**) ChitM and ChitM+G, (**C**) ChitH and ChitH+G membranes.

The characteristic peaks of chitosan are observed in each spectrum (Figure 3). The peak in the region 3402 cm^−1^ is from O-H and N-H present in the polysaccharide structure. The characteristic absorption peak at 2920 cm^−1^ is attributed to the C-H groups. The peak at 1710 cm^−1^ from C=O of chitosan is also observed. The presence of peaks from -NH at 1560 cm^−1^ and O-H at 1411 cm^−1^ are also present in each spectrum. The range 900–1200 cm^−1^ shows the band of peaks for the following bonds: C-C, C-O-C, C-O-H. Thereby, characteristic peaks of chitosan with low, medium and high molecular weight are observed in all membranes, with and without gentamicin addition [20]. There is no difference between the location of peaks; however, their intensity increases with increasing molecular weight of chitosan. The addition of gentamicin to the chitosan membranes did not affect the changes in the chitosan structure, as the location of peaks did not change (Figure 4). This suggests that gentamicin does not interact with chitosan and does not cross-link it. 

### 3.5. Mechanical Properties

**Figure 5:**
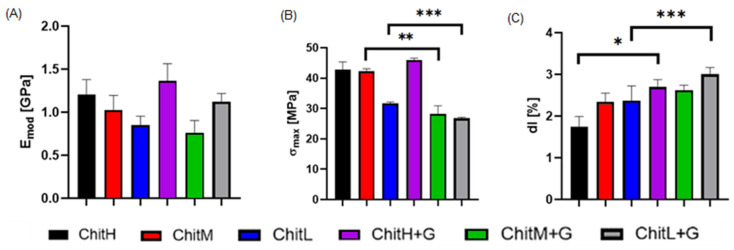
Mechanical properties of membranes determined in a static tensile test: (**A**) Young’s modulus, (**B**) maximum tensile strength and (**C**) elongation at break (n = 5; *—statistical significance between groups, *p* < 0.05; ** *p* < 0.01; *** *p* < 0.005). The molecular weight of chitosan influences the mechanical strength of the membranes.

The different molecular weight of chitosan affected the mechanical strength of the membranes; as it increased, the maximum tensile strength and Young’s modulus increased while elongation decreased. The addition of antibiotics to the membranes had different effects on their mechanical parameters. There was no significant impact on Young’s modulus. Maximum tensile strength decreased significantly in all membranes, with the exception of ChitH+G. In contrast, elongation at break improved in all cases (Figure 5A–C). Hence, the most favorable mechanical properties after the addition of the gentamicin showed membranes obtained from high-molecular-weight chitosan.

### 3.6. Cytocompatibility

**Figure 6:**
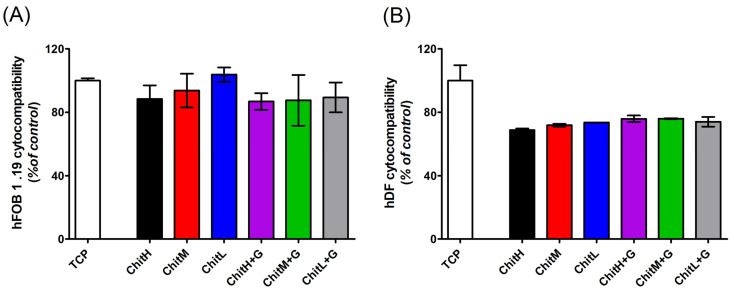
Cytocompatibility of membranes determined with (**A**) fFOB 1.19 cells (n = 4; *p* > 0.05) and (**B**) hDF cells (n = 2; *p* < 0.05) by MTT assay after 72 h of culture. The developed membranes were classified as cytocompatible.

The cytocompatibility of all developed membranes was tested on two cell lines: human osteoblasts: hFOB 1.19 and primary fibroblast derived from human skin: hDF. The effect was measured after 72 h of culture with different membrane types. The viability of the cells is expressed as a percentage of formazan reduction, which reflects mitochondrial activity. The control comprised the cells not treated by the membranes. The membranes did not affect the hFOB 1.19 viability (Figure 6A). There was a fall of about 15% in mitochondrial activity, independently of the chitosan used (Figure 6A). Moreover, low-molecular-weight chitosan (ChitL) increased the hFOB 1.19 viability (Figure 6A). These changes were not significant (Figure 6A). On the other hand, human dermal primary fibroblasts were more susceptible to membranes. High-molecular-weight chitosan (ChitH) caused a 30% fall in cellular viability, and the addition of the antibiotic to ChitH brought about a 25% fall in cell numbers compared to the non-treated control (TCP) (Figure 6B). The other tested membranes led to a 24% decrease in hDF viability. Therefore, the developed materials can be classified as cytocompatible. This is due to the fact the cellular viability in response to the tested membranes was above 75% (Figure 6A,B). 

### 3.7. Antibacterial Properties

**Figure 7:**
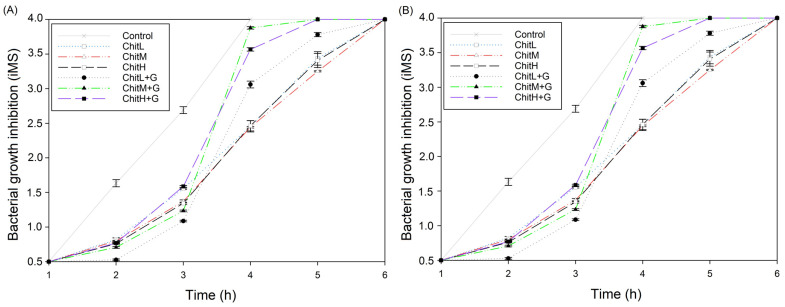
Antibacterial properties of membranes determined by McFarland index measuring the turbidity of bacteria broth: (**A**) *Staphylococcus aureus* and (**B**) *Escherichia coli* (n = 3; statistical significance compared to the control for all results—*p* < 0.05). The developed membranes slowed down the growth of bacteria.

**Figure 8:**
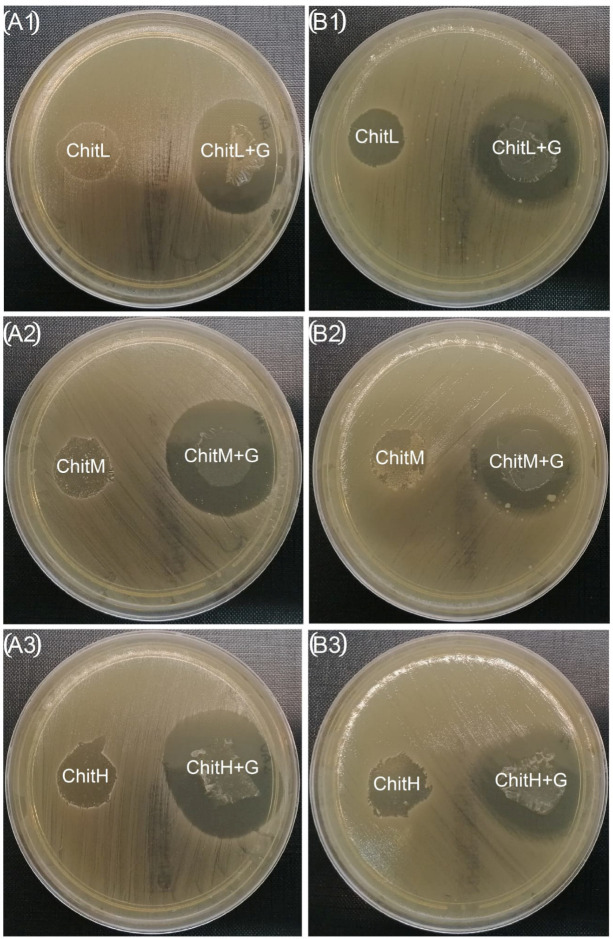
Antibacterial properties of membranes determined by the Kirby–Bauer zone of inhibition after seven days of incubation: (**A1**–**A3**) *Staphylococcus aureus* and (**B1**–**B3**) *Escherichia coli*. The presented pictures are representative of 3 experiments. Membranes with gentamicin showed a larger zone of inhibition.

All developed membranes showed antibacterial properties and slowed down the growth of both bacteria: *S. aureus* and *E. coli* in broth (Figure 7 and Figure 8, Table 2). Moreover, membranes with added gentamicin showed significant differences in antibacterial effectiveness compared to those without antibiotics. Greater effectiveness in inhibiting bacteria in the zone of inhibition test was found for chitosan with high molecular weight, especially after the addition of gentamicin (≈76% zone enlargement; Table 2). The zones kept their sizes unchanged for seven days (Figure 8). Furthermore, it was found that membranes reached larger inhibition zones for *S. aureus* than for *E. coli*. The study of the bacterial properties in the solution showed that, in the case of *S. aureus*, the highest effectiveness was observed for ChitH+G and ChitL+G, while ChitM+G showed worse activity than membranes without the addition of gentamicin (Figure 7A). However, in the case of *E. coli*, a different effect was observed and membranes without the antibiotic showed a more significant antibacterial effect after 3 h than membranes containing gentamicin. In the initial stage (up to 2 h), the highest effectivity against *E. coli* was observed for ChitL+G (Figure 7B).

## 4. Discussion

In this work, three types of chitosan membranes both with and without additional antibiotics were successfully obtained using a solvent casting method. The influence of the use of chitosan with different molecular weights (low, medium and high) on selected membrane properties, both physical and biological, was considered. The first difference regarding the use of chitosan with different molecular weights was visible during the fabrication process. The use of high- and low-molecular-weight chitosan was associated with problems in obtaining a homogeneous and compact membrane. The medium-molecular-weight chitosan was the most optimal for the proposed production method. Physicochemical changes in membranes made of chitosan with different molecular weights probably resulted from the fact that the degrees of chitosan crystallinity are also distinct, which is related to the degree of DD deacetylation and molecular weight. Modification of the degree of crystallinity alters the interactions present in the material because hydrogen interactions inside the molecule between the hydroxyl and amino groups of the chain are stronger than between these polar groups and water [13,20]. Additionally, the chitosan chain is ampliated in an aqueous solution, where it may create large aggregates [27,28], which consequently could affect the physicochemical properties of the membranes. The authors did not observe any qualitative changes in the viscosity or stickiness of the chitosan solutions in which gentamicin was added. Nonetheless, the presence of gentamicin may affect some alterations in the properties of membranes. This probably arises from the fact that the addition of gentamicin may reduce the viscosity of the solution, which results in fewer interactions between polymer and drug molecules through hydrogen bonds [29] and consequently on the characteristics of the membranes.

All obtained membranes have a roughness value in the range from 0.73 ± 0.11 µm to 10.96 ± 0.21 µm. The optimal roughness value of biomaterials, despite many studies, is still unidentified. However, Lukaszewska et al. [30] showed the advantage of a rough surface over smooth surfaces in terms of cell adhesion, colonization and proliferation. The benefit of moderately rough surfaces over significantly rough, minimally rough and smooth surfaces has also been proven in terms of bone regeneration processes [31]. The optimal roughness value increases the initiation and primary stability of the cells adjacent to the biomaterial [32]. In addition, an adequately adjusted value promotes the polarization of macrophages, consequently minimizing the occurrence of the inflammatory response [33]. The authors noticed that the addition of gentamicin had no effect on the roughness parameter for ChitL. On the other hand, in the case of ChitM, the addition of the antibiotic decreased the roughness of the film (from 10.96 ± 0.21 µm to 4.97 ± 0.10 µm), and in the case of ChitH increased it almost twofold (from 0.73 ± 0.11 µm to 1.39 ± 0.07 µm). This may be related to the fact that chitosan has the ability to retain a significant amount of foreign substances in its mass. The introduced compounds may bind with amino sugar residues, forming hydrogen, ionic and coordination bonds with them located on the -OH and -NH_2_ groups [34]. In this way, two- or large-component systems may be created in which chitosan acts as a carrier with a specific biological or biochemical activity [35]. In other words, chemical substances may be embedded in chitosan, changing the surface of the membranes and thus the roughness of the biomaterial. To sum up, there was no linear relationship that would indicate a change in roughness depending on the added antibiotic and chitosan molecular weight. In addition, the authors speculate that the applied method of membrane modification may be related to the high randomness of gentamicin deposition in the structure. However, it should not have a significant negative effect on the biological properties of developed membranes, as has been shown in studies. Furthermore, this randomness in the incorporation of an active substance, such as gentamicin, in various chitosan materials, contributes to differences in roughness, although data in the literature may show otherwise [36].

The contact angle measurements confirmed that all membranes possess a hydrophilic character, which can be induced by the presence of -OH groups in the chitosan side chains [37]. This may affect the antibacterial activity of the membranes. The hydrophilic nature of the biomaterial ensures the formation of a stable interfacial layer of water, which insulates it from direct contact with bacteria on the surface [38]. It was found that a different molecular weight of chitosan affects the value of the contact angle, and the highest value was noted for ChitM and thus this parameter cannot be linearly related. Zhong et al. [39] indicated that the contact angle increases with the increasing molecular weight of chitosan. However, previous studies concern chitosan with lower molecular weights (up to ≈50 kDa), while our research concerns chitosan with much higher molecular weights (50–190 kDa, 190–310 kDa and 310–375 kDa). Longer chitosan chains are more entangled, resulting in the formation of more inter- and intramolecular hydrogen bonds in the polysaccharide, which should have a positive effect on hydrophilicity. On the other hand, the drying process of the membranes may reduce the protonation of amino groups, consequently increasing the hydrophobic nature of chitosan chains [40]. Therefore, more research should be conducted in this area, focusing on these properties. Moreover, our study revealed that the addition of gentamicin did not significantly affect the wettability of the membranes. Due to the fact that biomaterial roughness significantly affects wettability [41] and gentamicin is highly hydrophilic [42], the authors expected differences in the values of contact angle results (roughness values of ChitL and ChitH coatings without and with antibiotic were different). However, it is possible that the two- or multi-component systems that may have formed after the addition of the antibiotic [35] had a similar nature to the membranes without gentamicin.

The FTIR spectra confirmed no significant changes in the chemical structure of membranes based on chitosan with different molecular weights with or without the addition of gentamicin. For all membranes, the characteristic peaks for chitosan were observed and only slight changes in their intensity were observed, without any shifts. The addition of gentamicin did not cause cross-linking of chitosan and was the only addition of actives in the matrix. No effect of different molecular weights on the structure of the obtained chitosan biomaterials was previously observed by Lieder et al. [6,9] in the production of chitosan membranes.

The mechanical properties are a vital application parameter for membranes designated as biomaterials. Nunthanid et al. [43] demonstrated that the mechanical strength of their films increased with an increase in the molecular weight of chitosan. The results obtained by us are similar, as the best mechanical properties, especially maximum tensile strength, were confirmed for the high-molecular-weight chitosan membranes. Simultaneously, the lowest elongation at break was obtained for this membrane. This may be due to the fact that chitosan of different molecular weights varies in the entanglement of the chitosan network that unfolds during axial stretching [44]. Additionally, it was reported by Bakhsheshi-Rad et al. [29] that the addition of gentamicin into the chitosan/alginate hydrogels significantly affects their mechanical parameters. It is in good agreement with our results, as we observed the decrease in maximum tensile strength and increased elongation at break. This phenomenon may be due to the fact that the antibiotic particles constitute a point of stress concentration in the polymer matrix, especially for low- and medium-molecular-weight chitosan membranes [45].

These studies show the lack of cytotoxic effect of all tested membranes on human osteoblastic cells and human fibroblasts. There were also no significant differences between the membranes based on chitosan with different molecular weights with or without the addition of gentamicin. Our results are consistent with previous observations on chitosan-based biomaterials. Hsu et al. [46] showed that molecular weight mainly affects the mechanical properties and the degradation rate of chitosan scaffolds, with no evident effect on fibroblasts L929 and chondrocytes IRC cytocompatibility. Additionally, Hamilton et al. [47] did not find a relationship between human bone cell NHOst cytocompatibility and chitosan film parameters: deacetylation degree, molecular weight and crystallinity. Furthermore, the beneficial effect of osteoconductive chitosan coatings on metallic implants was also confirmed [9]. Raza et al. [48] stated that high-molecular-weight chitosan sponges facilitate more effectively the secretion of factors stimulating bone remodeling (e.g., osteopontin or interleukin-6), Wimardhani et al. [49] discovered that low-molecular-weight chitosan had the potential to be a natural anticancer agent. Hence, it may be concluded that, regardless of the form of the chitosan biomaterial (coating, scaffold, sponge, etc.), it shows high cytocompatibility on osteoblastic cells. On the other hand, there was a trend of inhibition of primary dermal fibroblast viability. However, the strongest inhibition exerted ChitH and it was precisely 32%, therefore the cellular survival was above 60% compared to control conditions. This fall may result from the fact that primary cells tend to attach to negatively charged surfaces, and the membranes are neutral. Nevertheless, the insignificant fall indicates that developed membranes exert a weak cytostatic, not cytotoxic effect. Thus we may state that the developed membranes are cytocompatible. Our results are consistent with other data presented in the literature, where chitosan hydrogel was not toxic to primary mouse dermal cell proliferation [50]. Moreover, in this work, we confirmed that the addition of gentamicin does not adversely affect the biological properties of the chitosan membranes.

The membranes containing gentamicin showed antibacterial activity. In the experimental tests, it was confirmed that the antibiotic’s release from membranes might inhibit the multiplication of bacteria, both: *S. aureus* and *E. coli*, in broth and agar. Additionally, it was observed that chitosan itself has antibacterial potential but is more effective in a liquid environment. The antibacterial properties of chitosan, according to the most popular theory, are related to its positively charged active amino groups. This involves electrostatic interactions with the bacterial cells, which are negatively charged. As a result, disruption of microbial membrane integrity occurs. It is also assumed that chitosan may affect the osmotic balance of the membrane wall, block RNA and protein synthesis, or disturb the oxygen reduction process and electron transport. However, the exact bactericidal mechanisms of chitosan are not fully clarified yet [51]. Here, we found significant differences for chitosan with different molecular weights in terms of the antibacterial properties of membranes. In the zone of inhibition test, membranes obtained from high-molecular-weight chitosan with gentamicin showed the highest efficiency, especially for *S. aureus*, while in the bacterial growth inhibition test in solution, it was found that only ChitH and ChitL were suitable as carriers for gentamicin for *S. aureus*, which may be related to the fact that chitosan has the ability to retain a significant amount of foreign substances in its mass. For *E. coli*, the results were different, because the chitosan membrane itself (regardless of the molecular weight) showed better antibacterial activity tested in a liquid environment than the membrane with the addition of gentamicin. The antibacterial effectiveness of chitosan biomaterials has been tested before. Li et al. [52] described how low-molecular-weight chitosan might penetrate bacterial cells and inhibit RNA or protein synthesis as well as change the DNA conformation. However, Aranaz et al. [53] showed that an increase in molecular weight contributes to the improvement of permeation into the nucleus of bacteria. Furthermore, Li et al. [54] tested different kinds of chitosan fibers as antibacterial agents and found that increased molecular weight first increased and then decreased bactericidal activity of *S. aureus* and in the aspect of *E. coli* increasing only decreasing molecular weight antibacterial properties. These results are different from those obtained by us, but these differences may be related to the different forms of chitosan. Our research shows that the developed membranes are mainly effective against *S. aureus* and the use of high-weight chitosan would be recommended. For *E. coli*, the addition of gentamicin is not recommended, or it is recommended to reduce its content. The above differences may be related to the type of bacteria; *S. aureus* is a Gram-positive bacteria, while *E. coli* is Gram-negative. Additionally, as these bacteria have significant differences in their structure, this affects their resistance to antibacterial agents [55]. We assume that, in the case of *E. coli* in a liquid environment, the bacterium exposed to two harmful environmental agents (antibiotic and chitosan)formed a biofilm that protected it from the action of the membrane. This phenomenon has already been observed by Babu et al. [56], who studied the adsorption of chitosan on a solid support in contact with *E. coli*.

The parameters strongly affecting the properties of chitosan are the degree of deacetylation and its molecular weight [40], and to a large extent, the biological response of the produced chitosan forms will depend on these parameters. Here, we thoroughly studied the effect of different molecular weights of chitosan on selected properties of membranes obtained under the same conditions, with a particular focus on their use as carriers of antibiotics. In this study, we were able to show that high-molecular-weight chitosan membrane is the best carrier for gentamicin and that the gentamicin-loaded membranes (1% *w*/*w*) are only suitable for the treatment of *Staphylococcus aureus* infections. The addition of antibiotics did not negatively affect the chemical structure, wettability or cytocompatibility of the membranes, and, in the case of ChitH, there also was no reduction in mechanical properties.

## 5. Conclusions

In this study, membranes were successfully prepared from chitosan powder of different molecular weights. Three different types of chitosan membranes with and without gentamicin were proposed as cytocompatible biomaterials. Results indicate that the addition of gentamicin may change the topography and roughness of the chitosan membrane, whereas the chemical structure and wettability of membranes remained almost unchanged. The highest contact angle and roughness values were exhibited by the membrane fabricated from medium-molecular-weight chitosan. The most favorable mechanical properties of an antibiotic-loaded membrane are issued to a membrane prepared with high-molecular-weight chitosan. Antibacterial activity studies have shown that the high-molecular-weight chitosan is the most suitable carrier for gentamicin, and chitosan membranes are mainly effective for *S. aureus* infections. None of the prepared membranes showed a cytotoxic effect on osteoblastic or fibroblast cells; hence, they may be intended for various medical applications, i.e., as a drug carrier, an implant coating or a wound dressing.

## Figures and Tables

**Table 1:** The roughness of chitosan membranes (^#^ statistical significance between chitosan molecular weight; * statistical significance between chitosan with/without gentamicin, *p* < 0.05).

Membrane	R_a_ Mean [µm]
ChitL	1.57 ± 0.14 ^#^
ChitM	10.96 ± 0.21 ^#,^*
ChitH	0.73 ± 0.11 ^#,^*
ChitL+G	1.54 ± 0.16
ChitM+G	4.97 ± 0.10 *
ChitH+G	1.39 ± 0.07 *

**Table 2:** The Kirby–Bauer zone of inhibition test for chitosan membranes after 1, 3 and 7 days (n = 3; ^#^ statistical significance between chitosan molecular weight; * statistical significance between chitosan with/without gentamicin, *p* < 0.05).

Membrane	Zone of Inhibition (mm)	
	*Staphylococcus aureus*	*Escherichia coli*
ChitL	17 ± 1 ^#^	
ChitM	19 ± 1	
ChitH	20 ± 1 ^#^	
ChitL+G	34 ± 1 *	30 ± 1 *
ChitM+G	36 ± 1 *	31 ± 1 *
ChitH+G	38 ± 1 *	33 ± 1 *

## Data Availability

Data sharing not applicable.
